# Supplementary material for: Establishing the Competency Development and Talent Cultivation Strategies for Physician-Patient Shared Decision-Making Competency Based on the IAA-NRM Approach
Source: Healthcare (Basel). 2022 Sep 23;10(10):1844. doi: 10.3390/healthcare10101844 (PMC9601707; doi:10.3390/healthcare10101844)
Supplement: Supplementary file 1 [file healthcare-10-01844-s001.zip › healthcare-1901148-supplementary.pdf]

healthcare MDPI

Supplementary File

1. The IAA approach

The IAA for the current study is described below. The first adoption step was to enhance those aspects (i.e. PP, ES) within the third quadrant (L, L), indicating a low level of importance and awareness. The second adoption step is to improve those aspects (i.e., RI, SD) within the first quadrant (H, H), indicating the high importance degree and high awareness degree, as shown in Table S1 and Figure S1.

Table S1. The IAA (importance & awareness analysis) of SDM competency development.

Aspects	II		AI		(II, AI)
	MI	SI	MA	SA	
Perception assessment and practice (PP)	7.933	-0.565	7.673	-0.854	(L , L)
Execution process and skills (ES)	7.891	-1.092	7.681	-0.805	(L , L)
Physician-patient relationship and interaction (RI)	8.065	1.067	7.891	0.478	(H , H)
Shared information and decision making (SD)	8.026	0.590	8.006	1.181	(H , H)
Average	7.979	0.000	7.813	0.000	
Standard deviation	0.080	1.000	0.163	1.000	
Maximum	8.065	1.067	8.006	1.181	
Minimum	7.891	-1.092	7.673	-0.854	

Note 1: The first quadrant of (H, H) means high importance and high awareness; (L, H) means low importance and high awareness in the second quadrant. The third quadrant of (L, L) means low importance and low awareness, and (H, L) means high importance and low awareness in the fourth quadrant.
Note 2: MI, SI, MA, and SA mean importance level (MI), standardized importance level (SI), awareness level (MA), and standardized awareness level (SA), respectively.

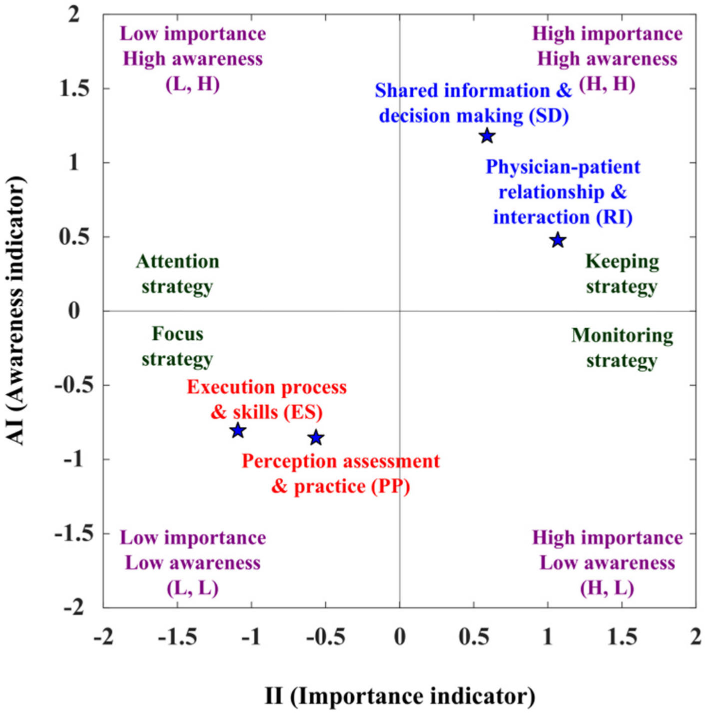

Figure S1. The analysis map of IAA (Importance & awareness analysis).

2. The DEMATEL approach

The DEMATEL approach involves five steps: (1) estimate the original average matrix; (2) compute the direct influence matrix; (3) compute the indirect influence matrix; (4) evaluate the full influence matrix; (5) examine the NRM relationship.

(1) The original average matrix

The original average matrix is as revealed in Table S2. The ES aspect's impact on the PP aspect is 2.653, which suggests "medium influence." The RI aspect's impact on the SD aspect is 2.726, which implies "medium influence."

Table S2. The original average influence matrix (*A*).

Aspects	PP	ES	RI	SD	Total
Perception assessment and practice (PP)	0.000	2.694	2.718	2.589	8.000
Execution process and skills (ES)	2.653	0.000	2.839	2.798	8.290
Physician-patient relationship and interaction (RI)	2.597	2.750	0.000	2.726	8.073
Shared information and decision making (SD)	2.565	2.669	2.774	0.000	8.008
Total	7.815	8.113	8.331	8.113	

(2) The direct influence matrix

From Equations (1) and (2), the *D* (direct influence matrix) can be attained by *A* (initial average influence matrix), as illustrated in Table S3. As indicated in Table S4, by counting the rows and columns, the sum of the ES and RI aspects ranks are 1.969, which are the most crucial affect aspects. The PP aspect is 1.898, the least influenced aspects.

Table S3. The direct influence matrix (*D*).

Aspects	PP	ES	RI	SD	Total
Perception assessment and practice (PP)	0.000	0.323	0.326	0.311	0.960
Execution process and skills (ES)	0.318	0.000	0.341	0.336	0.995
Physician-patient relationship and interaction (RI)	0.312	0.330	0.000	0.327	0.969
Shared information and decision making (SD)	0.308	0.320	0.333	0.000	0.961
Total	0.938	0.974	1.000	0.974	-

Table S4. The degree of direct influence.

Aspects	Sum of row	Sum of column	Sum of row and column	Importance of influence
Perception assessment and practice (PP)	0.960	0.938	1.898	4
Execution process and skills (ES)	0.995	0.974	1.969	1
Physician-patient relationship and interaction (RI)	0.969	1.000	1.969	1
Shared information and decision making (SD)	0.961	0.974	1.935	3

(3) The indirect influence matrix

The indirect influence matrix can be obtained from Equation (3) and presented in Table S5.

Table S5. The indirect influence matrix (*ID*).

Aspects	PP	ES	RI	SD	Total
Perception assessment and practice (PP)	7.994	8.141	8.311	8.155	32.601
Execution process and skills (ES)	8.136	8.445	8.534	8.369	33.484
Physician-patient relationship and interaction (RI)	7.977	8.197	8.452	8.207	32.832
Shared information and decision making (SD)	7.928	8.149	8.315	8.235	32.626
Total	32.034	32.932	33.612	32.965	-

(4) The full influence matrix

T (full influence matrix) and the degree of full influence can be attained by Equation (4) or (5) and presented in Table S6 and Table S7, respectively. The summation of rows (*d*) and the summation of columns (*r*) are calculated by using Equation (7) and (8). The RI aspect has the greatest full influence (d_3 + r_3= 68.413). The net influence is highest in aspect of PP (d_1 - r_1= 0.589). The order of other net effects is as follows: the ES aspect (d_2 - r_2= 0.574), SD (d_4 - r_4 = -0.352) and RI aspect (d_3 - r_3= -0.811).

Table S6. The full influence matrix (***T***).

Aspects	PP	ES	RI	SD	*d*
Perception assessment and practice (PP)	7.994	8.464	8.637	8.466	33.561
Execution process and skills (ES)	8.454	8.445	8.875	8.705	34.479
Physician-patient relationship and interaction (RI)	8.289	8.527	8.452	8.534	33.801
Shared information and decision making (SD)	8.236	8.469	8.648	8.235	33.587
r	32.972	33.905	34.612	33.939	-

Table S7. The degree of full influence.

Aspects	*d*	*r*	*d* + *r*	*d* - *r*
Perception assessment and practice (PP)	33.561	32.972	66.534	0.589
Execution process and skills (ES)	34.479	33.905	68.384	0.574
Physician-patient relationship and interaction (RI)	33.801	34.612	68.413	-0.811
Shared information and decision making (SD)	33.587	33.939	67.526	-0.352

(5) The NRM relationship.

As shown in Table S7, the X-axis and Y-axis can be drawn (d_i + r_i) and (d_i - r_i), respectively. The NRM graph can be devised, as indicated in Figure S2. The net influence matrix is illustrated in Table S8. The PP aspect is the dominate factor with net influence, while the RI aspect is the dominate factor being influenced. The RI aspect has the greatest full influence, while the PP aspect has the smallest full influence, as demonstrated in Figure S2 and Table S8. The PP aspect impacts the aspects of ES, SD, and RI. The ES aspect influences the aspects of SD and RI, and the SD aspect affects the RI aspect. Thus, the preferred strategic direction for developing SDM competency is enhancing PP.

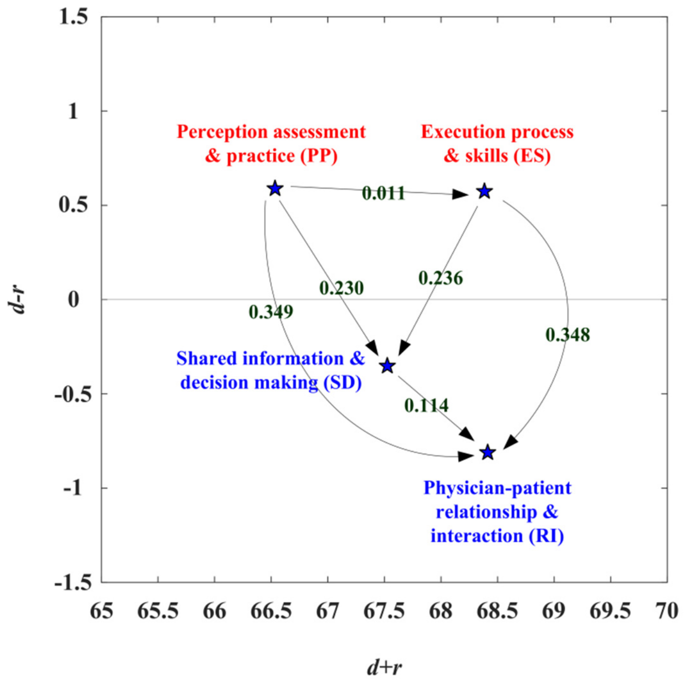

Figure S2. The NRM for SDM competency development.

Table S8. The net influence matrix for SDM competency development.

Aspects	PP	ES	RI	SD
Perception assessment and practice (PP)	-			
Execution process and skills (ES)	-0.011	-		
Physician-patient relationship and interaction (RI)	-0.349	-0.348	-	
Shared information and decision making (SD)	-0.230	-0.236	0.114	-
